# Phytochemicals from Brazilian Red Propolis: A Review of Their Anti-Inflammatory Potential

**DOI:** 10.3390/plants14192961

**Published:** 2025-09-24

**Authors:** Thaise Boeing, Rodolfo Moresco, Priscila de Souza

**Affiliations:** 1 Pharmaceutical Sciences Program, Universidade do Vale do Itajaí, Itajaí 88302-901, Santa Catarina, Brazil; 2 Biological Sciences Course, Universidade do Vale do Itajaí, Itajaí 88302-901, Santa Catarina, Brazil

**Keywords:** Brazilian red propolis, anti-inflammatory compounds, natural products

## Abstract

Brazilian red propolis (BRP) has emerged as a promising source of multifunctional phytochemicals with potent anti-inflammatory activity. This review provides a comprehensive analysis of the anti-inflammatory effects of BRP’s bioactive compounds, their molecular targets, and their mechanisms of action. Isolated compounds from BRP (such as formononetin, biochanin A, daidzein, calycosin, medicarpin, vestitol, and neovestitol) have demonstrated the ability to modulate critical pro-inflammatory signaling pathways, including NF-κB, TLR4, JAK/STAT, and PI3K/AKT, while concurrently activating antioxidant and cytoprotective responses via the Nrf2/HO-1 axis. These effects are further supported by the suppression of pro-inflammatory cytokines, regulation of immune cell infiltration and activation, inhibition of inflammasome components such as NLRP3, induction of autophagy, and polarization of macrophages and microglia from a pro-inflammatory (M1) to an anti-inflammatory (M2) phenotype. Collectively, these findings reinforce the potential of BRP as a rich source of multifunctional phytochemicals with broad therapeutic relevance for chronic inflammation and related pathologies. Future research should address the identified knowledge gaps by employing rigorous in vitro and in vivo toxicity assessments, exploring structure–activity relationships, and leveraging advanced delivery systems to optimize bioavailability. Such methodological approaches will be essential for translating the promising biological activities of BRP compounds into clinically viable therapeutic agents.

## 1. Introduction

Brazilian red propolis (BRP) is a unique type of propolis predominantly produced in the northeastern region of Brazil. It has gained significant scientific and commercial interest due to its rich and diverse chemical composition and potent biological activities, particularly its anti-inflammatory effects [1]. Propolis, a resinous substance collected by bees from various plant sources, serves as a natural defense mechanism against microbial infections and environmental threats in the hive [2]. The bioactive compounds present in BRP contribute to its pharmacological properties, making it a valuable natural product for therapeutic applications.

The primary botanical source of BRP has been identified as *Dalbergia ecastaphyllum* (L.) Taub. (Fabaceae), which provides isoflavonoids and other phenolic compounds [3,4]. However, recent studies have highlighted the presence of additional bioactive compounds, such as polyprenylated benzophenones, whose origin has been linked to *Symphonia globulifera* L.f. (Clusiaceae) [5]. These diverse phytochemicals, including flavonoids, triterpenoids, and benzophenones, contribute to BRP’s pharmacological potential, particularly in modulating inflammatory pathways.

Inflammation is a complex biological response involved in various pathological conditions, including chronic diseases such as arthritis, cardiovascular disorders, neurodegenerative diseases, gastrointestinal illnesses, and cancer [6]. Natural products, such as those derived from BRP, have been extensively studied for their role in modulating inflammatory mediators, reducing oxidative stress, and influencing immune responses [7].

This review aims to explore the anti-inflammatory properties of BRP by analyzing its phytochemical composition and the molecular mechanisms by which its bioactive compounds exert their biological effects. By compiling current findings from in vitro and in vivo studies, this work seeks to provide comprehensive insights into the therapeutic potential of BRP’s compounds for the development of novel anti-inflammatory agents.

## 2. Chemical Composition of BRP and Its Plant Source

BRP has a distinctive red color, and its chemical profile is directly related to its botanical origin, primarily the leguminous plant *D. ecastophyllum* (commonly known as *rabo-de-bugio*), which grows in mangrove ecosystems. The resinous exudates collected by bees from *D. ecastophyllum* are rich in isoflavonoids and other phenolic compounds, which differentiate BRP from other types of propolis produced worldwide. Formononetin was the most abundant compound in both propolis and resin [4]. Other important bioactive compounds identified include biochanin A, vestitol, neovestitol, isoliquiritigenin, liquiritigenin, calycosin, medicarpin, and daidzein [4,8,9,10,11]. In addition to these constituents, compounds such as rutin, quercetin, luteolin, ferulic acid, chrysin, pinobanksin, and pinocembrin [3,8,12] have also been identified, albeit in lower concentrations. According to the study by Ccana-Ccapatinta et al. [5], *S. globulifera* was identified for the first time as a botanical source of polyprenylated benzophenones, notably guttiferone E and oblongifolin B, in addition to the triterpenoids β-amyrin and glutinol.

**Figure 1:**
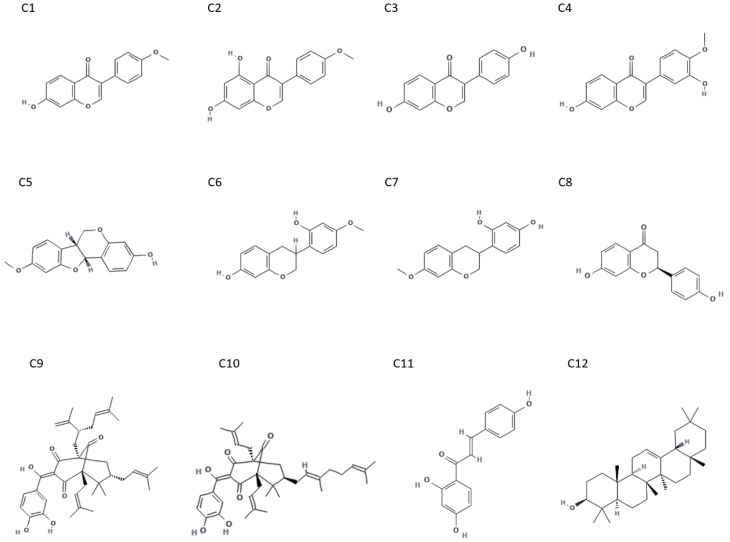
Chemical structures of the main bioactive compounds identified in BRP. Formononetin (C1), biochanin A (C2), daidzein (C3), calycosin (C4), medicarpin (C5) vestitol (C6), neovestitol (C7), liquiritigenin (C8), guttiferone E (C9), oblongifolin B (C10), isoliquiritigenin (C11), and β-amyrin (C12).

Given their well-documented biological activities and consistent presence in BRP, the compounds formononetin [7-hydroxy-3-(4-methoxyphenyl)chromen-4-one], biochanin A [5,7-dihydroxy-3-(4-methoxyphenyl)chromen-4-one], daidzein [7-hydroxy-3-(4-hydroxyphenyl)chromen-4-one], calycosin [7-hydroxy-3-(3-hydroxy-4-methoxyphenyl)chromen-4-one], medicarpin [(6aR,11aR)-9-methoxy-6a,11a-dihydro-6H-[1]benzofuro[3,2-c]chromen-3-ol], vestitol [3-(2-hydroxy-4-methoxyphenyl)-3,4-dihydro-2H-chromen-7-ol], neovestitol [4-(7-methoxy-3,4-dihydro-2H-chromen-3-yl)benzene-1,3-diol], liquiritigenin [(2S)-7-hydroxy-2-(4-hydroxyphenyl)-2,3-dihydrochromen-4-one], guttiferone E [(1R,3E,5S,7S)-3-[(3,4-dihydroxyphenyl)-hydroxymethylidene]-6,6-dimethyl-5,7-bis(3-methylbut-2-enyl)-1-[(2R)-5-methyl-2-prop-1-en-2-ylhex-4-enyl]bicyclo[3.3.1]nonane-2,4,9-trione], oblongifolin B [(1R,3E,5S,7R)-3-[(3,4-dihydroxyphenyl)-hydroxymethylidene]-7-[(2E)-3,7-dimethylocta-2,6-dienyl]-6,6-dimethyl-1,5-bis(3-methylbut-2-enyl)bicyclo[3.3.1]nonane-2,4,9-trione], isoliquiritigenin [(E)-1-(2,4-dihydroxyphenyl)-3-(4-hydroxyphenyl)prop-2-en-1-one], and β-amyrin [(3S,4aR,6aR,6bS,8aR,12aR,14aR,14bR)-4,4,6a,6b,8a,11,11,14b-octamethyl-1,2,3,4a,5,6,7,8,9,10,12,12a,14,14a-tetradecahydropicen-3-ol]—chemical structures are depicted in Figure 1—were selected for in-depth analysis in this review. These molecules stand out not only for their significant contribution to the anti-inflammatory, antioxidant, and antimicrobial properties of BRP but also for representing the most structurally diverse and pharmacologically relevant chemical classes within its composition, including isoflavonoids, polyphenols, benzophenones, and triterpenes. This selection enables a more focused discussion of their mechanisms of action, offering valuable insights for their potential development into therapeutic agents.

## 3. Anti-Inflammatory Potential of BRP Compounds

BRP is a rich source of bioactive phytochemicals known for their diverse pharmacological properties, particularly their anti-inflammatory effects. This section explores the anti-inflammatory potential of key compounds isolated from BRP, providing a comprehensive review of their mechanisms of action, efficacy, and therapeutic relevance. Each topic is dedicated to one or more compounds (grouped by similarity), discussing their chemical characteristics, reported biological activities, molecular targets involved in inflammation modulation, and insights from in vitro and in vivo studies. Through this detailed organization, the section aims to highlight the promising contributions of BRP compounds in the development of novel anti-inflammatory therapies.

### 3.1. Formononetin

Formononetin, a plant-derived isoflavone, has been identified and quantified as a major chromatographic marker (21.62 mg/g) in the hydroalcoholic extract of BRP [13,14]. Substantial evidence has consistently demonstrated its potent anti-inflammatory properties. In rodent models, both the hydroalcoholic extract of red propolis at doses of 10 and 30 mg/kg and purified formononetin at 10 mg/kg effectively alleviated visceral and inflammatory pain, as shown by reduced paw edema and leukocyte migration [13].

More recently, formononetin has been shown to exert strong anti-inflammatory and antiproliferative effects by modulating interferon (IFN)-mediated immune responses in psoriasiform inflammation. In cultured HaCaT keratinocytes, formononetin significantly reduced cell viability (IC50 = 40.64 µM) and suppressed the production of tumor necrosis factor-alpha (TNF-α) and interleukin-6 (IL-6), with maximal effects observed at 20 µM. In an animal model of psoriasis induced by imiquimod, topical application of a 2% formononetin cream for 12 days resulted in a significant improvement of skin lesions, characterized by reduced epidermal thickness, scaling, erythema, and a decrease in Psoriasis Area and Severity Index (PASI) scores. These effects were associated with inhibition of the IFN signaling pathway, evidenced by downregulation of IFN-α, IFN-β, IFN-γ, TNF-α, IL-17, IFN-related chemokines (Cxcl9, Cxcl10, Cxcl11, Cxcr3), and phosphorylated signal transducer and activator of transcription 1 (STAT1), STAT3, and interferon regulatory factor (IRF)1 [15].

In macrophages stimulated with lipopolysaccharide and in a zebrafish inflammation model, formononetin at 20 µM significantly reduced IL-6 and IL-1β levels and inhibited macrophage recruitment at inflammatory sites. Mechanistically, formononetin promoted autophagy (upregulation of LC3II/I, downregulation of P62) and induced macrophage polarization from the pro-inflammatory M1 phenotype (CD86-positive) to the anti-inflammatory M2 phenotype (CD206-positive), likely through modulation of TNF, epidermal growth factor receptor, prostaglandin-endoperoxide synthase 2, and estrogen receptor signaling pathways [16].

In models of polycystic ovary syndrome induced by dehydroepiandrosterone in rats and by dihydrotestosterone in human granulosa-like tumor cells, oral administration of formononetin at doses of 15, 30, and 60 mg/kg attenuated symptoms by reducing oxidative stress, inflammation, and apoptosis. These beneficial effects were attributed to the suppression of NLR family pyrin domain-containing 3 (NLRP3) inflammasome activation, both in animals and cell cultures [17].

In the cardiovascular field, formononetin demonstrated significant protection against myocardial ischemia and reperfusion injury in rats. Administered at 20 mg/kg, formononetin improved cardiac function, decreased infarct size, and reduced platelet activation, neutrophil extracellular trap formation, and microthrombus accumulation. These effects were linked to the downregulation of cluster of differentiation 36 (CD36) and inhibition of extracellular signal-regulated kinase 5 phosphorylation, leading to reduced platelet–neutrophil interactions and inflammatory responses. Another study further supported these findings, showing that oral administration of formononetin at 20 mg/kg reduced myocardial damage, improved echocardiographic parameters, and suppressed expression of TNF-α and nuclear factor kappa B (NF-κB). Moreover, sequencing of the 16S ribosomal RNA gene revealed that formononetin modulated the gut microbiota by increasing the abundance of anti-inflammatory genera such as *Ligilactobacillus* and *Blautia* and reducing the abundance of pro-inflammatory genera such as *Treponema*, suggesting that part of its anti-inflammatory effect is mediated through modulation of the gut microbiota–NF-κB axis [18].

Additionally, in an in vitro model of thermal injury using human skin fibroblasts (HSFs), formononetin (10 µM) promoted cell proliferation and migration while reducing apoptosis, ROS production, and inflammatory cytokine levels (IL-1β and TNF-α). These effects were mediated through activation of the PI3K/AKT/mTOR signaling pathway, reversing injury-induced downregulation of its phosphorylated components [19]. Similarly, in a cell-based model of neuroinflammation using oxygen-glucose deprivation followed by reperfusion in microglial BV2 cells, formononetin (10 µM) inhibited pro-inflammatory microglial activation. It significantly decreased IL-1β and TNF-α production, blocked nuclear translocation of the p65 subunit of NF-κB, and downregulated TLR (toll-like) 4, p-IκBα, and p65 expression. Formononetin also promoted M2 polarization of microglia, underscoring its neuroprotective and anti-inflammatory potential through modulation of the TLR4/NF-κB signaling pathway [20].

Taken together, the studies reviewed consistently demonstrate that formononetin possesses robust anti-inflammatory activity across a wide range of experimental models, both in vitro and in vivo. Effective concentrations in vitro typically range from 10 to 40 µM, while in vivo models show therapeutic doses generally between 10 and 60 mg/kg, depending on the disease context. Mechanistically, formononetin acts through multiple converging pathways that regulate inflammation, including inhibition of pro-inflammatory cytokines (such as TNF-α, IL-1β, and IL-6), suppression of key inflammatory signaling cascades (e.g., IFN/STAT, TLR4/NF-κB, and PI3K/AKT/mTOR), modulation of immune cell polarization (e.g., shifting macrophages and microglia from M1 to M2 phenotype), and attenuation of inflammasome activation (e.g., NLRP3). These findings suggest that formononetin exerts its anti-inflammatory effects not through a single target but rather via a multifaceted mechanism, supporting its potential as a promising therapeutic candidate for treating various inflammation-related diseases.

Beyond its anti-inflammatory activity, both red propolis and formononetin have demonstrated antioxidant and gastroprotective effects [21], further supporting their role in protecting tissues against inflammation-induced damage. Formononetin is now recognized as one of the primary bioactive constituents responsible for many of the therapeutic properties of red propolis, including its immunomodulatory effects. Interestingly, while both the crude extract and pure formononetin effectively reduced inflammatory pain, the extract occasionally showed superior efficacy—likely due to synergistic effects from other active compounds present in the mixture [1].

However, despite encouraging preclinical data, several limitations must be acknowledged. Formononetin faces significant challenges for therapeutic use due to its poor oral bioavailability (21.8%), rapid absorption, and short half-life [22,23]. These limitations are attributed to its low water solubility and fast clearance. While generally considered safe, high doses can cause adverse effects, such as transient vomiting in dogs at 300 mg/kg, and mild liver changes in mice at high doses. The NOAEL (No-Observed-Adverse-Effect Level) was 100 mg/kg in dogs [24]. Therefore, despite its safety at therapeutic levels, its limited bioavailability and dose-dependent toxicity require further pharmacological optimization and more extensive safety studies for its development as a drug. Thus, while promising, formononetin remains at an early stage of the drug development pipeline, requiring further evidence before clinical application can be realistically considered.

### 3.2. Biochanin A

Biochanin A is an isoflavone with estrogenic activity and is classified as a phytoestrogen, capable of interacting with estrogen receptors and mimicking or modulating their effects in key physiological processes, thus being considered a potential endocrine disruptor. Despite concerns regarding its estrogen receptor (ER) activity in hormone-dependent cancers, recent evidence indicates that it may also exert antitumor effects. Wang et al. [25] demonstrated that biochanin A induces apoptosis in ER^+^ MCF-7 breast cancer cells through inhibition of the PI3K/AKT pathway, without stimulating ER-mediated proliferation. These findings underscore its complex pharmacological profile and therapeutic potential in selected hormone-responsive tumors. Biochanin A demonstrates potent antileukemic activity in acute myeloid leukemia (AML) by inducing dose-dependent apoptosis through caspase-7 activation, PARP1 cleavage, and suppression of oncogenic drivers, while upregulating pro-apoptotic and cell cycle inhibitory mediators. These multi-targeted actions position biochanin A as a promising therapeutic candidate for AML, particularly in TP53-mutant subtypes reliant on p53-independent apoptotic pathways [26].

Biochanin A demonstrated both anti-inflammatory and antifibrotic effects in bleomycin-induced pulmonary fibrosis models. The compound inhibited the TGF-β1/Smad2/3 pathway, reducing epithelial–mesenchymal transition (EMT), inflammatory cell infiltration, and collagen deposition, suggesting a role in modulating tissue remodeling [27]. Similarly, Zhang et al. [28] evaluated biochanin A in an experimental model of periodontitis, administering intravenous doses of 12.5, 25, and 50 mg/kg/day over four weeks. Biochanin A significantly reduced proinflammatory cytokines (IL-1β, TNF-α), reactive oxygen species (ROS), and alveolar bone loss, while enhancing Nrf2 and osteocalcin expression, thereby demonstrating local antioxidant and anti-inflammatory effects.

In dermatological models, formulations of biochanin A (0.3%, 1%, and 3%) attenuated imiquimod-induced psoriasis-like lesions in mice, suppressing cytokines such as IL-17A and IL-23, and downregulating NF-κB and MAPK pathways [29]. Lv et al. [30] corroborated these findings using in vitro and in vivo models, showing that biochanin A reduced inflammatory mediators (COX-2, iNOS, ILs) and enhanced IL-10 and antioxidant enzymes via activation of the Nrf2/HO-1 axis.

Feng et al. [31] reported that intraperitoneal administration of biochanin A (25 and 50 mg/kg/day) protected against transverse aortic constriction (TAC)-induced cardiac hypertrophy, reducing fibrosis, inflammation, and collagen secretion. Similarly, Mahajan et al. [32] demonstrated the cardioprotective effects of oral biochanin A (5–20 mg/kg) in a diabetic myocardial infarction model, with improved hemodynamic parameters, reduced inflammatory cytokines, and increased antioxidant defenses.

In gastrointestinal inflammation, Deng et al. [33] showed that biochanin A restored intestinal barrier integrity and inhibited ferroptosis by downregulating the JAK2/STAT3 pathway and modulating GPX4 in DSS-induced colitis. Ram et al. [34] also reported beneficial effects in unilateral ureteral obstruction-induced renal fibrosis, with inhibition of TGF-β1/Smad2/3 and NF-κB/NLRP3 signaling and induction of the Nrf2/HO-1 pathway.

Li et al. [35] demonstrated neuroprotective effects of biochanin A (40 mg/kg, intraperitoneally, for 14 days) in a spinal cord injury model. The compound attenuated inflammation, pyroptosis, and oxidative stress via inflammasome inhibition and activation of Nrf2/HO-1 and autophagy-related pathways. In the oncological setting, Thakkar et al. [36] identified biochanin A as an inhibitor of glucose-6-phosphate dehydrogenase (G6PD) in A549 non-small-cell lung cancer cells, with an IC50 of 21.92 µM. Biochanin A downregulated the expression of IL-6, IL-8, MMP-2, and MMP-9 and exhibited anti-inflammatory and antimetastatic activity, comparable to diclofenac in in vitro assays.

Collectively, the evidence reviewed positions biochanin A as a multifunctional compound with consistent anti-inflammatory activity mediated by the inhibition of classical inflammatory pathways and activation of antioxidant responses. Its well-documented pharmacological profile underscores its potential as a promising natural agent for the development of novel therapeutic strategies targeting chronic inflammatory diseases. However, despite its wide-ranging pharmacological actions, biochanin A remains limited by significant translational gaps. Most evidence stems from preclinical models, with scarce clinical validation to confirm its efficacy or safety in humans. Its dual phytoestrogenic activity raises concerns regarding hormone-dependent cancers, where it may exert both protective and proliferative effects depending on tumor context and estrogen receptor status.

### 3.3. Daidzein

Daidzein, an isoflavonoid compound, is a key chemical constituent present in the hydroalcoholic extract of BRP, where it has been quantified as 4.68 µg/mL or 19.90 mg/g. This compound, along with other flavonoids and isoflavonoids in propolis, significantly contributes to the extract’s diverse biological activities, including its notable antioxidant and anti-inflammatory properties. Specifically, the hydroalcoholic extract of BRP, containing daidzein, has demonstrated chemoprotective and anti-inflammatory effects in vivo. Its anti-inflammatory activity is linked to a reduction in leukocyte migration and the release of pro-inflammatory cytokines. The extract also exhibits significant antioxidant activity, which is crucial for its photoprotective and chemopreventive properties [37,38].

In vitro, daidzein mitigates lipopolysaccharide (LPS)-induced hepatocyte injury by suppressing inflammation and oxidative stress. At a concentration of 100 µM, daidzein significantly reduced the expression of inflammatory mediators IL-1β (73.8% ± 5.3%), IL-6 (58.8% ± 9.0%), and TNF-α (55.5% ± 7.2%) in LPS-treated hepatocytes, primarily via inhibition of the ERK1/2 and NF-κB signaling pathways [39]. Furthermore, daidzein reduced LPS-induced reactive oxygen species (ROS) production and increased superoxide dismutase (SOD) activity by downregulating Keap-1 and upregulating Nrf2 expression [40].

In vivo studies in mice have demonstrated that daidzein alleviates doxorubicin (DOX)-induced heart failure by attenuating cardiac inflammation, oxidative stress, apoptosis, and fibrosis. Daily intraperitoneal administration of daidzein (10 mg/kg) significantly improved cardiac function and normalized DOX-induced alterations in serum and cardiac levels of IL-1β, TNF-α, NLRP3, IL-6, and MCP-1. In vitro studies with H9c2 cardiomyoblasts confirmed that daidzein protects against DOX-induced cell injury through the SIRT3/FOXO3a signaling pathway, by reducing oxidative stress, inflammation, and apoptosis [41].

In sows, dietary supplementation with daidzein (200 mg/kg) improved reproductive performance by modulating ovarian oxidative stress and inflammatory responses. This intervention significantly decreased malondialdehyde (MDA), IL-1β, IL-6, and TNF-α levels in ovarian tissue, while enhancing the activities of total antioxidant capacity (T-AOC) and catalase (CAT). The underlying mechanism involves inhibition of the TLR4/NF-κB signaling pathway and activation of the Nrf2/HO-1 axis, as evidenced by decreased expression of TLR4, phosphorylated NF-κB, AKT, and IκBα, alongside increased levels of Nrf2, HO-1, and NQO1 [42].

In an ex vivo model using LPS-stimulated murine peritoneal macrophages, daidzein (100 µM) and its metabolite equol (25 µM) effectively suppressed inflammatory responses. Both compounds reduced COX-2 expression, prostaglandin E2 (PGE2) levels, nitrite production, and inducible nitric oxide synthase (iNOS) expression. Additionally, they inhibited canonical inflammasome signaling by downregulating NLRP3 protein expression and subsequently decreasing IL-1β and IL-18 levels. Molecular docking studies support the modulation of NF-κB and direct binding of daidzein to COX-2, iNOS, and NLRP3 as potential mechanisms underlying these effects [43]. In contrast, in a 6-month randomized, double-blind, placebo-controlled trial, Liu et al. [44] investigated the effects of whole soy (soy flour) and purified daidzein on bone turnover and inflammatory markers in 270 equol-producing postmenopausal Chinese women. Participants were assigned to receive either 40 g of soy flour (containing 49.8 mg isoflavones), 40 g of milk powder plus 63 mg of purified daidzein, or a placebo (40 g of milk powder) daily. The trial found no statistically significant differences among the three groups in changes or percentage changes in key bone turnover markers, including serum β-CTX, PINP, osteocalcin, and bone-specific alkaline phosphatase, or inflammatory markers such as IL-6, TNF-α, hs-CRP, transferrin, and homocysteine. These findings suggest that neither whole soy nor isolated daidzein supplementation at the tested dosages significantly affects bone metabolism or systemic inflammation in this specific population.

Daidzein has also shown neuroprotective properties in vitro, mitigating amyloid-β (Aβ)-induced cytotoxicity in SH-SY5Y neuroblastoma and C6 glioma cells. Pretreatment with daidzein (0.5 µM) preserved cell viability, attenuated oxidative stress, and maintained mitochondrial membrane potential. It also reduced the levels of MAPK pathway proteins (JNK, p-JNK, p38), pro-inflammatory mediators (COX-2, IL-1β), and pyroptosis markers (caspase-1, gasdermin D). These effects are attributed to modulation of the JNK pathway and suppression of neuroinflammation [45].

In models of nephrotoxicity, daidzein exerted protective effects against gentamicin-induced renal damage both in vitro (MDCK cells) and in vivo (zebrafish). It suppressed oxidative stress, inflammation, and apoptosis in MDCK cells, and in zebrafish, daidzein (100 µM) significantly decreased COX-2, TNF-α, and IL-1β levels. Moreover, it enhanced antioxidant defenses by increasing SOD and glutathione (GSH) levels while reducing lipid peroxidation and nitric oxide (NO) production [46].

Finally, daidzein showed renoprotective effects in a rat model of glycerol-induced acute kidney injury (AKI). Oral administration of daidzein (100 mg/kg) significantly reduced IL-1β, TNF-α, myeloperoxidase (MPO), and NF-κB levels, while enhancing the anti-inflammatory cytokine IL-10. It also increased antioxidant biomarkers (SOD, CAT, GPx, GR, GSH), upregulated the expression of Nrf2 and Hmox1 genes, and modulated apoptosis by reducing Bax and caspase-3 levels and increasing Bcl-2. Molecular docking supports interactions of daidzein with Keap1-Nrf2, NF-κB, and caspase-3 pathways [47].

Overall, the evidence highlights daidzein as a multifunctional bioactive compound with significant anti-inflammatory, antioxidant, and cytoprotective properties across a variety of experimental models. Its ability to modulate key molecular signaling pathways—such as NF-κB, Nrf2, SIRT3/FOXO3a, and TLR4—underlies its therapeutic potential in mitigating inflammation, oxidative stress, and apoptosis in hepatic, cardiac, renal, ovarian, and neural tissues. These findings support the pharmacological relevance of daidzein, not only as a major constituent of BRP but also as a promising candidate for the development of novel therapeutic strategies targeting inflammation-related pathologies.

Despite the relevant biological findings, some points should be considered. Daidzein demonstrates considerable acute oral toxicity, with a median lethal dose (LD50) of 150 mg/kg. Nevertheless, research has shown that administration of daidzein at doses up to 1000 mg/kg does not cause significant alterations in hematological parameters, biochemical markers, or kidney function, although minor changes in serum glucose, lipid profiles, and relative organ weights were observed. Computational toxicity predictions indicate that daidzein carries a low risk for mutagenicity, carcinogenicity, immunotoxicity, hepatotoxicity, skin irritation, reproductive toxicity, hERG channel inhibition, and toxicity to aquatic organisms [48,49]. Additionally, supplementation with soy isoflavones, which contain daidzein, in both male and female rats led to only slight, non-adverse changes in liver enzymes and other biochemical parameters, reinforcing the compound’s wide safety margin [50].

### 3.4. Calycosin

Calycosin has been extensively studied for its pharmacological properties, particularly its anti-inflammatory activity. Numerous in vitro and in vivo studies have demonstrated its effects across a variety of pathological models, often involving modulation of classical inflammatory signaling pathways such as NF-κB, TLR4, and HIF-1α.

Calycosin’s neuroprotective effect was demonstrated by Yang et al. [51], who used a middle cerebral artery occlusion model in rats. Intraperitoneal administration of calycosin one-hour post-ischemia significantly reduced neurological deficits, infarct volume, and tissue inflammation. Complementary in vitro studies using PC12 cells and primary neurons subjected to oxygen and glucose deprivation further revealed that calycosin inhibited the HMGB1/TLR4/NF-κB pathway, leading to reduced expression of IL-6 and IL-18.

Liu et al. [52,53] investigated the anti-fibrotic and anti-inflammatory potential of calycosin in bleomycin-induced pulmonary fibrosis in mice. Oral administration at 7 and 14 mg/kg reduced inflammation, apoptosis, and collagen deposition. Mechanistically, calycosin modulated the AKT/GSK3β/β-catenin pathway and promoted autophagy and antioxidant defenses via the Nrf2/HO-1 axis, indicating multifaceted protective effects in fibrotic lung injury.

The anti-inflammatory scope of calycosin also extends to dermatological disorders. In a murine model of atopic dermatitis, Ma et al. [54] showed that topical application of calycosin significantly decreased pro-inflammatory cytokines (IL-4, IL-5, IL-13, TSLP) and shifted the immune balance toward regulatory T cells (Tregs) by increasing FOXP3 expression and suppressing Th17 differentiation markers (IL-17A, RORγt), suggesting its potential for immune regulation in skin inflammation.

Gastrointestinal applications were reported by Peng et al. [55], who evaluated the effects of calycosin (20, 40, and 80 mg/kg) in rats subjected to gastrectomy-induced mucosal injury. Calycosin ameliorated systemic inflammation (TNF-α, IL-6, IL-1β), oxidative stress, and bacterial translocation, while enhancing tight junction integrity via upregulation of occludin and ZO-1. These findings support its role in preserving epithelial barrier function under inflammatory stress.

Further corroborating its systemic anti-inflammatory profile, Wang et al. [56] investigated the effects of calycosin in a rat model of post-myocardial infarction heart failure. Oral treatment (80 mg/kg for 28 days) led to significant reductions in TNF-α, IL-6, and cardiac fibrosis. In vitro, calycosin suppressed PI3K–AKT and IKK/NF-κB activation in H9C2 cardiomyocytes and TGF-β-stimulated fibroblasts, confirming a dual anti-inflammatory and anti-fibrotic mechanism. Neuroprotective mechanisms were explored by Xu et al. [57], who employed the MCAO model in rats and an OGD/R model in PC12 cells. Calycosin at 30 mg/kg decreased infarct size and serum TNF-α and IL-6 levels. Additionally, it downregulated autophagy-related markers through inhibition of the STAT3/FOXO3a signaling pathway, thereby mitigating oxidative stress and neuronal apoptosis.

Finally, in the context of osteoarthritis, Shi et al. [58] demonstrated that calycosin, at doses ranging from 100 to 400 µM in vitro and 40 mg/kg intraperitoneally in vivo, suppressed IL-1β-induced production of IL-6, TNF-α, iNOS, and COX-2 in chondrocytes. It also inhibited cartilage matrix degradation and apoptosis via downregulation of the PI3K/AKT and NF-κB pathways, suggesting chondroprotective properties.

Taken together, these findings consistently demonstrate the anti-inflammatory efficacy of calycosin across a spectrum of experimental models, ranging from neural and cardiac tissues to the skin, intestine, and joints. Key molecular targets include NF-κB, TLR4, CXCL10, STAT3, and PI3K/AKT, with effective doses spanning from 10 to 100 µM in vitro and 7 to 80 mg/kg in vivo. To date, no clinical trials have been reported investigating calycosin’s anti-inflammatory effects in humans, underscoring the need for translational research. Pharmacokinetic studies indicate that calycosin exhibits favorable absorption and distribution characteristics. Nanoliposomal formulations have been shown to enhance its bioavailability and therapeutic efficacy, particularly in cancer treatment models [59]. Well-designed clinical trials will be essential to establish its therapeutic value and guide its future clinical applications.

### 3.5. Medicarpin

Medicarpin is a pterocarpan widely found in BRP, particularly derived from the plant *D. ecastophyllum*. Its complex phytochemical profile has attracted scientific interest due to its broad spectrum of pharmacological properties, including antimicrobial, antioxidant, antiproliferative, and anti-inflammatory activities.

One of the most well-documented actions of medicarpin is its ability to modulate the cellular antioxidant response, which is closely linked to the regulation of inflammation. Kim et al. [60] demonstrated that medicarpin activates the NRF2 pathway in HeLa cells, enhancing the transcription of antioxidant-related genes. According to the authors, “medicarpin significantly induced the antioxidant response elements (ARE)-luciferase activity in a concentration-dependent manner,” and further, it “strongly inhibited the ubiquitin-dependent proteasomal degradation of NRF2.” As NRF2 plays a central role in the response to oxidative and inflammatory stress, its activation by medicarpin supports the compound’s role as an indirect anti-inflammatory modulator.

Boeing et al. [61] investigated the gastroprotective effects of red propolis and its isolated constituents, including medicarpin, in a murine model of ethanol/HCl-induced gastric ulcers. The study reported that “MD (10 mg/kg p.o.) protected gastric mucosa against the damage induced by ethanol/HCl,” and that this effect was associated with reduced oxidative stress and myeloperoxidase (MPO) activity—an important inflammatory biomarker. Additionally, “its effect was abolished by indomethacin treatment,” suggesting that the protective mechanism involves prostaglandin pathways.

Chen et al. [62] assessed the effects of medicarpin on bladder cancer cell lines (T24 and EJ-1). While the focus was on its antiproliferative capacity, the study also pointed to indirect anti-inflammatory effects. The authors noted that “MED significantly upregulated pro-apoptotic proteins BAK1, Bcl2-L-11, and caspase-3,” and “suppressed tumor growth in vivo” in murine models. The regulation of mitochondrial apoptotic pathways—often activated during chronic inflammation—suggests a broader role for medicarpin in modulating inflammation-associated tumorigenesis.

More recently, Squarisi et al. [63] evaluated a benzophenone-free red propolis extract (BFRP), with medicarpin identified as one of its major constituents, in a rat model of colon carcinogenesis. The results were promising: “Chemoprevention studies showed a 41.6% reduction in preneoplastic lesions in rats treated with 6 mg/kg of BFRP,” with no genotoxic or cytotoxic effects observed up to a dose of 2000 mg/kg. Although the study’s primary aim was chemoprevention, the reduction in inflammatory lesions suggests that medicarpin may help modulate the intestinal inflammatory microenvironment.

In addition to its effects on oxidative and inflammatory cells, medicarpin has demonstrated selective antimicrobial activity, which could indirectly influence inflammation of infectious origin. Williams et al. [64] reported that “(+)-medicarpin selectively inhibited *Neisseria* gonorrhoeae with a minimum inhibitory concentration value of 0.25 mg/mL.” This selective action against pathogenic microorganisms, with no apparent adverse effects, raises the possibility that medicarpin may exert secondary immunomodulatory activity.

The evidence indicates that medicarpin exhibits a multifaceted anti-inflammatory effect, involving activation of the NRF2 pathway, modulation of prostaglandin synthesis, and inhibition of neutrophil infiltration. These mechanisms, observed at concentrations ranging from 2.5 to 10 µM in vitro and 10 mg/kg in vivo, underscore its therapeutic potential, particularly considering its favorable safety profile in experimental models. Nevertheless, despite the promising preclinical data, most studies remain limited to experimental settings, and clinical or translational research is still scarce. Moreover, while its selective antimicrobial activity is noteworthy, its relevance in real-world inflammatory infectious conditions requires further validation. Therefore, although medicarpin emerges as a compound with significant pharmacological promise, its clinical applicability hinges on a more comprehensive understanding of its molecular mechanisms, pharmacokinetics, and long-term safety. It is worth noting that medicarpin exhibited selective cytotoxicity against the HeLa cell line [65], and further studies are needed to balance efficacy with potential toxicological risks.

### 3.6. Vestitol and Neovestitol

Vestitol and neovestitol are characteristic isoflavonoids of red propolis, widely recognized as chemical markers for quality control and botanical authentication, predominantly derived from *D. ecastaphyllum* [1,8]. HPLC and NMR analyses confirm their consistent presence in both red propolis extracts and the plant resin, with vestitol showing particularly high concentrations in the resin (5.615 g/100 g of dried material) [8].

Both compounds exhibit notable anti-inflammatory properties. In the study by Bueno-Silva et al. [9], intraperitoneal administration of vestitol or neovestitol (10 mg/kg) in murine models significantly reduced neutrophil migration, with efficacy comparable to dexamethasone (1 mg/kg). These effects were linked to the modulation of inflammatory mediators, decreased leukocyte rolling and adhesion, and, in the case of neovestitol, nitric oxide (NO)-dependent mechanisms [9,66]. In vitro, vestitol (0.55 μM) inhibited NO production and reduced pro-inflammatory cytokines such as GM-CSF, IL-6, TNF-α, IL-1β, and IL-1α, while increasing IL-10. These effects involved NF-κB and Erk 1/2 pathway inhibition and the induction of an M2 macrophage phenotype in LPS-activated cells [67]. Vestitol also downregulated genes associated with inflammation and tissue destruction (Icam-1, Wnt5a, Mmp7) while upregulating anti-inflammatory regulators (Socs3, Dab2) [68].

Neovestitol has also demonstrated immunomodulatory activity in both acute and chronic inflammation models. In LPS-induced peritonitis and collagen-induced arthritis, it reduced neutrophil infiltration, ICAM-1 expression, and IL-6 production, while sparing IL-17 pathways and Th17 cell populations, suggesting selective cytokine regulation [66].

Beyond anti-inflammatory effects, neovestitol exhibits anticancer potential. Nani et al. [69] reported that in HeLa cells it selectively inhibited prostaglandin E synthase (PGES), a gene associated with inflammation-driven tumor progression, whereas vestitol modulated genes linked to cytoskeletal structure and chromatin regulation. Aldana-Mejía et al. [70] demonstrated selective cytotoxicity of neovestitol against various human cancer cell lines (SK-MEL, SK-OV-3, KB, BT-549), with IC_50_ values ranging from 15.40 to 18.13 μg/mL, and no toxicity toward normal cell lines (VERO, LLC-PK1), indicating a favorable therapeutic window. At the molecular level, both isoflavonoids converge on NF-κB inhibition and suppression of pro-inflammatory mediators, including IL-1β, IL-6, TNF-α, COX-2, and PGES [63].

Regarding gastrointestinal protection, Boeing et al. [61] found that neovestitol alone produced only a modest reduction of ethanol/HCl-induced gastric lesions in mice and did not significantly affect mucin secretion, oxidative stress markers, or myeloperoxidase activity, unlike methylvestitol and medicarpin. This suggests that its gastroprotective potential may depend on synergistic interactions within the red propolis phytocomplex.

Overall, vestitol and neovestitol stand out as multifunctional phytochemicals with therapeutic potential in inflammatory and neoplastic diseases, capable of modulating critical signaling pathways and showing selective cytotoxicity toward tumor cells. These findings support their candidacy as natural lead compounds for the development of novel anti-inflammatory and anticancer agents and warrant further investigation into their pharmacodynamics and molecular targets. Regarding their safety profile, preliminary data suggest that vestitol and neovestitol possess low toxicity. Acute and sub-acute oral toxicity studies of Brazilian red propolis, which contains these compounds, have not reported significant adverse effects in rats, indicating a relatively safe profile for oral administration [71]. Nevertheless, the specific contributions of vestitol and neovestitol to these outcomes require further investigation. Comprehensive toxicological studies are essential to fully assess their safety profiles, especially for potential therapeutic applications.

### 3.7. Isoliquiritigenin and Liquiritigenin

Isoliquiritigenin and liquiritigenin are structurally related flavonoids identified as major phenolic constituents of BRP, occurring in both Amazonian and *D. ecastaphyllum*-derived varieties. Their presence, confirmed by chromatographic and spectrometric analyses, underscores their dual role as bioactive compounds and reliable chemical markers for extract standardization [5,8,72,73].

Isoliquiritigenin, a chalcone-type flavonoid, exhibits potent anti-inflammatory activity by inhibiting NF-κB, MAPK (ERK, JNK, p38), Notch1, and JAK2/STAT3 pathways, leading to suppression of TNF-α, IL-1β, IL-6, iNOS, and COX-2 in LPS-stimulated macrophages [74]. It confers protection against inflammatory and oxidative injury in various disease models, including diabetic nephropathy, where the chalcone (10–20 μM) reduced inflammatory, fibrogenic, and apoptotic markers via SIRT1 activation, MAPK/p38 suppression, and Nrf2/HO-1 upregulation [75], and multiple sclerosis, in which doses of 50–200 mg/kg modulated immune cell populations and promoted neuroprotective astrocyte phenotypes [76]. In cancer, isoliquiritigenin-rich BRP extracts reduced DMH-induced preneoplastic colorectal lesions by 41.6% [63], while in pancreatic cancer, it induced apoptosis through autophagy blockade mediated by p38 MAPK, with synergistic effects alongside standard chemotherapies [77].

Liquiritigenin, a flavanone, has demonstrated antiparasitic, anti-inflammatory, and organ-protective properties. In silico and in vitro analyses revealed strong binding affinity to *Leishmania* CYP51 (−9.3 kcal/mol) and TR (−8.9 kcal/mol), correlating with leishmanicidal activity and low macrophage toxicity [72]. It is also quantitatively confirmed as a BRP marker in both propolis and *D. ecastaphyllum* leaves and resin [9]. Liquiritigenin reduces TNF-α and IL-6 in LPS-stimulated macrophages, alleviates collagen-induced arthritis by suppressing NF-κB p65 [78], and protects cartilage integrity by inhibiting MMPs in IL-1β-stimulated chondrocytes [79]. In rheumatoid arthritis with cardiac involvement, it improves joint and cardiac function by downregulating pro-inflammatory cytokines, MMP-3/MMP-13, and TGF-β1/Smad2/3 signaling [80]. Mechanistic studies show that liquiritigenin elevates intracellular cAMP in dendritic and T cells (10–50 μM), modulating adenylyl cyclase and phosphodiesterase expression to suppress pro-inflammatory cytokines and shift T-cell polarization [81]. In Alzheimer’s disease models, it shifts microglia from M1 to M2 phenotypes, reduces NLRP3 and cleaved caspase-1, and improves cognitive performance [82]. Additionally, it mitigates arsenic trioxide-induced liver injury by reducing oxidative stress and inflammation via mTOR-mediated autophagy activation [83].

Together, isoliquiritigenin and liquiritigenin exhibit complementary bioactivities— anti-inflammatory, antioxidant, immunomodulatory, antiparasitic, and anticancer—through modulation of key molecular pathways, including NF-κB, MAPK, JAK/STAT, TGF-β1/Smad, Nrf2/HO-1, cAMP, and mTOR. Their natural abundance in BRP and broad pharmacological profile position them as promising candidates for drug development. Future research should prioritize clinical validation, pharmacokinetic optimization, and formulation strategies to enhance bioavailability and therapeutic efficacy in humans.

Previous studies have shown that isoliquiritigenin exhibits dose-dependent developmental toxicity in zebrafish embryos, leading to heart, liver, and nervous system malformations, including pericardial edema, yolk retention, and body shape abnormalities. These effects are associated with increased ROS levels and apoptosis via the Nrf2-HO1/JNK-ERK mitochondrial pathway [84]. Conversely, isoliquiritigenin formulated as zein-phosphatidylcholine hybrid nanoparticles demonstrated safety in acute and subacute toxicity studies in mice and rats. No mortality or significant changes in biochemical, hematological, or histopathological parameters were observed, suggesting that the nanoparticle formulation may mitigate toxicity [85].

While isoliquiritigenin and liquiritigenin possess promising therapeutic potential, their toxicity profiles differ. Isoliquiritigenin’s toxicity appears to be formulation-dependent, with nanoparticle encapsulation enhancing safety. In contrast, liquiritigenin has demonstrated protective effects against various toxicities. These findings underscore the importance of considering both the compound and its formulation when evaluating safety for therapeutic applications.

### 3.8. Guttiferone E and Oblongifolin B

Guttiferone E and Oblongifolin B are prominent polyprenylated benzophenones present in BRP, a resinous substance collected by *Apis mellifera* bees from plant exudates, mainly from *D. ecastaphyllum* and *S. globulifera* [5,86]. These benzophenones have been identified as major bioactive constituents of BRP, contributing to its anti-inflammatory, antimicrobial, and cytotoxic activities. Quantitative analysis has shown that guttiferone E together with its isomer xanthochymol represents approximately 16.68% of a benzophenone-rich extract from red propolis, while oblongifolin B can constitute up to 42.25% of this same extract [87].

Guttiferone E has been extensively studied both as part of BRP extracts and in its isolated form. In a chemopreventive study using a rodent model of colon carcinogenesis, a hydroalcoholic extract of red propolis containing guttiferone E was administered orally at doses of 12, 24, and 48 mg/kg. The extract significantly reduced the expression of cyclooxygenase-2 (COX-2) and proliferating cell nuclear antigen (PCNA) in colon tissue of rats exposed to 1,2-dimethylhydrazine (DMH), indicating anti-inflammatory and antiproliferative effects [88]. However, guttiferone E was not evaluated in isolation in that model.

A more recent study investigated isolated guttiferone E in osimertinib-resistant non-small-cell lung cancer (NSCLC) cells (H1975 line) and xenograft models. Guttiferone E was administered both alone and in combination with carboplatin. In vitro, it reduced cell viability with IC_50_ values of 2.56 µM in 2D cultures and 11.25 µM in 3D cultures. In vivo, intraperitoneal administration of 10 mg/kg significantly reduced tumor volume. Guttiferone E downregulated several inflammation- and survival-associated pathways, including mTOR, SIRT1, SIRT7, COX-2, and PD-L1, demonstrating its potential in inflammation-driven carcinogenesis [89].

Furthermore, guttiferone E demonstrated immunomodulatory and antimicrobial activity against methicillin-resistant *Staphylococcus aureus* (MRSA). At concentrations of 10–25 µM, the compound showed direct antimicrobial effects and enhanced the bactericidal capacity of immunosuppressed THP-1-derived macrophages, without significantly altering TNF-α or IL-6 production. These findings suggest that guttiferone E may support innate immune responses without triggering excessive inflammation, a hallmark of effective anti-inflammatory agents [90].

Oblongifolin B, another major benzophenone in BRP, has also shown promising biological activity. In studies on breast cancer cell lines, oblongifolin B exhibited cytotoxicity with IC_50_ values of 31.46 µM (MCF-10A), 17.88 µM (MCF-7), and 18.37 µM (MDA-MB-231), indicating a degree of selectivity for malignant cells [87]. Additionally, oblongifolin B significantly reduced clonogenic survival and increased the frequency of interphase nuclei in cancer cells, suggesting interference with cell cycle progression and potential anti-inflammatory action within the tumor microenvironment.

In a separate study, oblongifolin B was tested alongside guttiferone E for activity against MRSA strains isolated from wound infections. Both compounds demonstrated strong antibacterial effects and were capable of inhibiting biofilm formation [90]. Although the direct impact of these compounds on inflammatory mediators has not been fully characterized, their ability to reduce bacterial load and disrupt biofilms underscores their potential relevance in controlling infection-associated inflammatory responses.

Guttiferone E and oblongifolin B are two of the most abundant and pharmacologically active benzophenones found in BRP. Both compounds have demonstrated noteworthy anti-inflammatory properties, either through direct inhibition of molecular mediators such as COX-2 and mTOR, or indirectly via cytostatic, immunomodulatory, or antimicrobial actions. The concentration range tested for these compounds in vitro was approximately 2.5 µM to 31 µM, and in vivo doses ranged from 10 to 48 mg/kg. Although further mechanistic and clinical studies are needed, current evidence positions these natural benzophenones as promising candidates for the development of novel anti-inflammatory and immunomodulatory agents, particularly in the context of chronic inflammation, infection, and inflammation-driven cancer.

### 3.9. β-Amyrin

β-amyrin is a pentacyclic triterpene with well-documented anti-inflammatory, antioxidant, and immunomodulatory properties. Multiple studies have confirmed and expanded its therapeutic relevance in models of inflammation, fibrosis, and neurodegeneration. In the study conducted by Thirupathi et al. [91], the effects of β-amyrin were evaluated in a murine model of carbon tetrachloride (CCl_4_)-induced hepatic fibrosis. Oral administration of β-amyrin at doses of 25 and 50 mg/kg, three times per week for six weeks, resulted in a significant reduction in pro-inflammatory cytokines such as TNF-α, IL-1β, and IL-6, indicating strong anti-inflammatory activity in the liver. Moreover, β-amyrin attenuated collagen deposition and the expression of fibrotic markers, including α-SMA and TGF-β1, thereby demonstrating relevant anti-fibrotic effects. The compound also modulated hepatic apoptosis by decreasing pro-apoptotic proteins (caspase-3 and Bax) and increasing the expression of the anti-apoptotic protein Bcl-2. These findings suggest that β-amyrin possesses multifunctional therapeutic potential against chronic liver injury, acting simultaneously on inflammatory, fibrotic, and apoptotic pathways.

In a study by Askari et al. [92], the anti-inflammatory effects of β-amyrin were evaluated in lipopolysaccharide (LPS)- and interferon-gamma (IFN-γ)-stimulated microglial cells, an established in vitro model for central nervous system inflammation. Treatment with β-amyrin at concentrations of 12, 25, and 50 µM significantly suppressed the production of pro-inflammatory cytokines (TNF-α, IL-1β, IL-6, and PGE2) and downregulated inflammatory enzymes (iNOS and COX-2). Furthermore, β-amyrin enhanced the expression of arginase-1 (Arg1), indicative of M2 microglial polarization, which is associated with anti-inflammatory and tissue repair responses. The compound also exhibited a favorable safety profile, showing no cytotoxicity at concentrations up to 100 µM and promoting cell viability under inflammatory conditions.

Zahid et al. [93] evaluated the anti-inflammatory and anticancer potential of β-amyrin in a nickel chloride-induced colon cancer model in albino rats. Animals received oral β-amyrin at a dose of 2.5 mg/kg body weight for one month following a 12-week exposure to nickel (1 mg/kg/week, intraperitoneally). The treatment resulted in a moderate reduction in inflammatory and oxidative stress biomarkers, such as carbonyl protein (7.26 µmol/mL), creatinine (4.01 mg/L), and 8-OHdG (31.25 pg/mL), when compared to the positive control group. There was also an improvement in antioxidant enzyme levels, including SOD (0.85 IU/mL), GSH (3.26 µmol/mL), and CAT (1.65 µmol/mL). Molecular docking analysis revealed strong binding affinity of β-amyrin with VEGF (−8.7 kcal/mol), IL-10 (−9.1 kcal/mol), and MMP-9 inhibitor (−8.0 kcal/mol), indicating significant interactions with proteins involved in angiogenesis and tumor progression. While β-amyrin alone was effective, its combination with β-sitosterol and epiafzelechin produced more pronounced effects, suggesting a synergistic interaction in reducing inflammation and promoting colonic tissue recovery.

Cai et al. [94] investigated the protective effects of β-amyrin on bleomycin (BLM)-induced pulmonary fibrosis in mice. Intragastric administration of β-amyrin at doses of 20, 40, and 80 mg/kg significantly reduced pulmonary inflammation, as evidenced by decreased serum levels of IL-6, IL-1β, TNF-α, and MCP-1. Additionally, the compound exerted marked antioxidant effects, with increased activities of SOD, GPX, and GSH, along with decreased malondialdehyde (MDA) levels, indicating reduced tissue oxidative damage. β-amyrin also improved pulmonary function, as shown by arterial blood gas analysis, and reduced collagen deposition in the lungs, as assessed by histological staining and hydroxyproline content. These findings support the protective role of β-amyrin against pulmonary fibrosis through modulation of oxidative stress and inflammatory responses, positioning it as a promising candidate for the development of natural product-based antifibrotic therapies.

β-amyrin shows great promise for pharmaceutical development due to its anti-inflammatory, antioxidant, anti-fibrotic, and anticancer activities. Its effectiveness in both monotherapy and combined treatments makes it a strong candidate for managing chronic diseases. Further research is needed to improve its bioavailability and support its use in advanced therapeutic formulations.

Table 1 provides a systematic overview of the bioactive compounds identified in Brazilian red propolis, summarizing their anti-inflammatory activities, experimental models, molecular targets, and therapeutic implications. This comprehensive framework contextualizes the pharmacological relevance of BRP within inflammation research and serves as a valuable reference for future investigations.

## 4. Research Methodology

In this review, a comprehensive literature search was conducted using the following databases: PubMed, Scopus, and Web of Science. The review encompassed all regions and time periods, with article selection finalized in June 2025. The keywords applied in the search strategy included “Brazilian red propolis”, “inflammation”, “anti-inflammatory activity”, and the specific bioactive compounds or plant name. The inclusion criteria encompassed original research articles, review papers, books, and book chapters published in English, Spanish, or Portuguese, specifically addressing the chemical composition of Brazilian red propolis and its bioactive compounds with anti-inflammatory potential. Studies involving the isolated compounds and evaluation of biological activity in in vitro, in vivo, or in clinical models were prioritized. The exclusion criteria were articles without full-text access, conference abstracts without full data, and non-peer-reviewed materials such as dissertations or theses.

**Figure 2:**
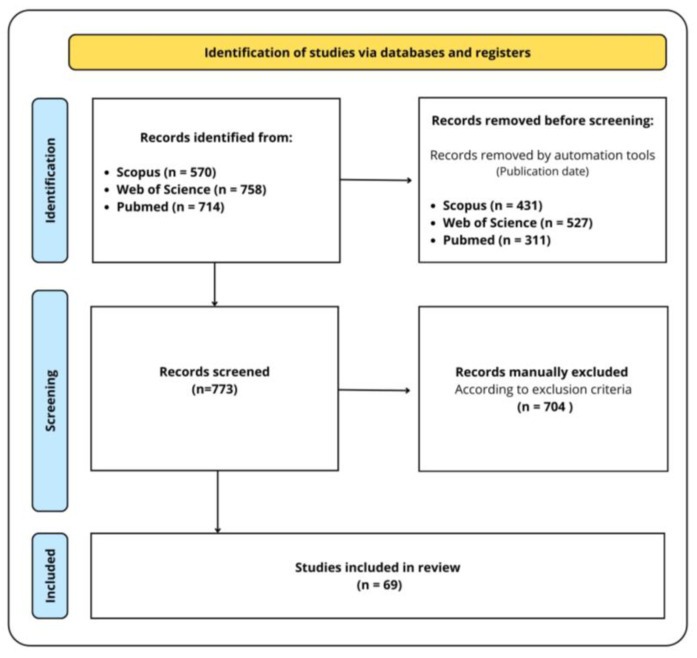
Selection process of the eligible reports based on the PRISMA 2020 flow diagram.

This review was conducted in accordance with the Preferred Reporting Items for Systematic Reviews (PRISMA) guidelines. The selection process for the papers included in this review is illustrated in Figure 2.

## 5. Conclusions and Future Remarks

This review compiles the growing body of evidence regarding the anti-inflammatory potential of phytochemicals from BRP. The compounds modulate major pro-inflammatory signaling pathways, including NF-κB, TLR4, JAK/STAT, and PI3K/AKT, and activate antioxidant responses via the Nrf2/HO-1 axis. Their effects involve suppression of cytokine production, inflammasome inhibition, immune cell reprogramming (M1 to M2), and enhancement of autophagy. In summary, BRP represents a promising source of multifunctional phytochemicals with potential against chronic inflammation and related diseases.

The major criticism of the compounds studied in this review is the clear lack of clinical studies, which makes it difficult to translate the promising preclinical findings into safe and effective human therapies. The future direction lies in overcoming this gap, requiring the immediate advancement of well-delineated clinical trials, especially for compounds that have already demonstrated promising safety and efficacy profiles in animal models. Concurrently, research should continue to focus on the pharmacological optimization of the compounds and the development of new formulations, with the ultimate goal of maximizing bioavailability and efficacy, thereby ensuring that the vast anti-inflammatory potential of these phytochemicals is validated and translated into clinical benefits.

Some compounds, such as isoflavones, can benefit from clinical studies focused on other purposes. Biochanin A, daidzein, and formononetin, for instance, have already been investigated in clinical trials. Randomized trials have demonstrated that isoflavone supplementation can improve bone mineral density and reduce bone reabsorption markers in postmenopausal women, supporting its role in osteoporosis prevention. Additionally, isoflavone-rich extracts containing combinations of these compounds have been associated with improvements in lipid metabolism, reductions in LDL cholesterol, and enhanced vascular endothelial function, suggesting cardioprotective potential [95,96]. While these compounds are generally considered safe and well-tolerated, clinical outcomes remain heterogeneous, and their efficacy appears to depend on factors such as dosage, treatment duration, and individual differences in gut microbiota.

In conclusion, the promising path forward lies not just in identifying new compounds but in a dedicated effort to validate their efficacy and safety in human trials, ultimately unlocking their therapeutic promise.

## Figures and Tables

**Table 1:** Experimental evidence of anti-inflammatory effects of BRP compounds.

Compound	Study Type	Experimental Model	Species/Cell Line	Concentration/Dose	Key Findings	Reference
Formononetin	In vivo	Inflammatory and visceral pain model	Mouse	10–30 mg/kg (extract), 10 mg/kg (pure)	Reduced paw edema; leukocyte migration.	[13]
	In vitro	HaCaT keratinocytes (psoriasiform inflammation)	Human	20–40 µM	Decreased TNF-α, IL-6, epidermal thickness via IFN/STAT pathway inhibition.	[15]
	In vivo	Imiquimod-induced psoriasis	Mouse	2% topical cream, 12 days	Reduced PASI scores, epidermal thickness, pro-inflammatory cytokines.	[15]
	In vitro	LPS-stimulated macrophages, zebrafish inflammation	Murine, zebrafish	20 µM	Reduced IL-6, IL-1β; promoted autophagy and M1-to-M2 shift.	[16]
	In vivo	PCOS induced by DHEA	Rat	15–60 mg/kg	Reduced oxidative stress, inflammation, NLRP3 activation.	[17]
	In vivo	Myocardial ischemia–reperfusion injury	Rat	20 mg/kg	Reduced infarct size, platelet activation, neutrophil traps via CD36/ERK5 inhibition.	[18]
	In vitro	Human skin fibroblasts (thermal injury)	Human	10 µM	Increased proliferation/migration; reduced apoptosis and cytokines.	[19]
	In vitro	BV2 microglia (OGD/R)	Murine	10 µM	Reduced TNF-α, IL-1β; inhibited TLR4/NF-κB; induced M2 polarization.	[20]
Biochanin A	In vivo	Bleomycin-induced pulmonary fibrosis	Mouse	5–10 mg/kg	Inhibited TGF-β1/Smad2/3; reduced inflammation and fibrosis.	[27]
	In vivo	Experimental periodontitis	Rat	12.5–50 mg/kg/day, i.v., 4 weeks	Reduced IL-1β, TNF-α, ROS; increased Nrf2 and osteocalcin.	[28]
	In vivo	Imiquimod-induced psoriasis	Mouse	0.3–3% topical	Reduced IL-17A, IL-23; inhibited NF-κB/MAPK.	[29]
	In vitro *&* in vivo	Various inflammatory models	Murine, rat	5–50 mg/kg	Increased IL-10, antioxidant enzymes via Nrf2/HO-1 activation.	[30]
	In vivo	DSS-induced colitis	Mouse	40 mg/kg	Restored barrier integrity, inhibited ferroptosis via JAK2/STAT3 modulation.	[33]
	In vivo	Renal fibrosis (UUO model)	Mouse	40 mg/kg	Inhibited TGF-β1/Smad2/3 and NF-κB/NLRP3.	[34]
	In vivo	Spinal cord injury	Rat	40 mg/kg i.p., 14 days	Reduced inflammasome activation, oxidative stress; activated Nrf2/HO-1.	[35]
	In vitro	NSCLC A549 cells	Human	IC50 = 21.92 µM	Reduced IL-6, IL-8, MMPs; anti-metastatic effect.	[36]
Daidzein	In vitro	LPS-treated hepatocytes	Human	100 µM	Reduced IL-1β, IL-6, TNF-α via ERK1/2 and NF-κB inhibition.	[39]
	In vivo	DOX-induced heart failure	Mouse	10 mg/kg i.p.	Reduced cardiac inflammation, oxidative stress, fibrosis.	[41]
	In vivo	Ovarian oxidative stress (sows)	Pig	200 mg/kg in diet	Decreased MDA, IL-1β, IL-6, TNF-α via TLR4/NF-κB inhibition.	[42]
	Ex vivo	LPS-stimulated peritoneal macrophages	Mouse	100 µM	Reduced COX-2, PGE2, iNOS; inhibited NLRP3 inflammasome.	[43]
	In vivo	Gentamicin nephrotoxicity	Zebrafish	100 µM	Reduced COX-2, TNF-α, IL-1β; increased antioxidants.	[46]
	In vivo	Acute kidney injury	Rat	100 mg/kg p.o.	Reduced TNF-α, MPO, NF-κB; increased IL-10 and antioxidants.	[47]
Calycosin	In vivo	MCAO-induced stroke	Rat	5–20 mg/kg	Reduced infarct size, IL-6, IL-18 via HMGB1/TLR4/NF-κB inhibition.	[51]
	In vivo	Pulmonary fibrosis (bleomycin)	Mouse	7–14 mg/kg p.o.	Reduced inflammation, collagen deposition via Nrf2/HO-1.	[52,53]
	In vivo	Atopic dermatitis	Mouse	Topical	Reduced IL-4, IL-5, IL-13; increased Tregs (FOXP3+).	[54]
	In vivo	Gastrectomy-induced injury	Rat	20–80 mg/kg p.o.	Reduced TNF-α, IL-6; improved tight junctions.	[55]
	In vivo	Post-MI heart failure	Rat	80 mg/kg p.o.	Reduced TNF-α, IL-6, fibrosis; inhibited PI3K/AKT and NF-κB.	[56]
	In vitro *&* in vivo	Osteoarthritis	Rat, chondrocytes	100–400 µM in vitro; 40 mg/kg i.p.	Reduced IL-1β, TNF-α, MMPs; protected cartilage.	[58]
Medicarpin	In vitro	HeLa cells	Human	50–100 µM	Activated NRF2; increased antioxidant gene expression.	[60]
	In vivo	Ethanol/HCl-induced gastric ulcer	Mouse	10 mg/kg p.o.	Reduced oxidative stress, MPO activity; prostaglandin-dependent.	[61]
	In vitro	Antiproliferative	T24, EJ-1 cells	50–100 µM	Upregulated pro-apoptotic proteins (BAK1, Bcl2-L-11, caspase-3); indirect anti-inflammatory effects.	[62]
	In vivo	Chemoprevention	Rat	6 mg/kg BFRP; No genotoxicity up to 2000 mg/kg	Reduced preneoplastic lesions; modulation of intestinal inflammatory microenvironment.	[63]
Vestitol	In vivo	Peritonitis model	Mouse	10 mg/kg i.p.	Reduced neutrophil migration; modulated cytokines.	[9]
	In vitro	LPS-macrophages	Murine	0.55 µM	Reduced NO, IL-6, TNF-α; increased IL-10; NF-κB inhibition.	[67]
Neovestitol	In vivo	Peritonitis, arthritis models	Mouse	10 mg/kg i.p.	Reduced neutrophil infiltration, IL-6; NO-dependent.	[9,66].
	In vitro	LPS-macrophages	Murine	0.3–30 µM	Reduced ICAM-1, IL-6; preserved IL-17/Th17.	[66]
Isoliquiritigenin	In vitro	LPS-macrophages	Murine	10–20 µM	Inhibited NF-κB, MAPK; reduced TNF-α, IL-1β, IL-6.	[74]
	In vitro	Diabetic nephropathy model	Murine cells	10–20 µM	Reduced inflammatory, fibrogenic, and apoptotic markers via SIRT1 activation, MAPK/p38 suppression, and Nrf2/HO-1 upregulation.	[75]
	In vivo	Multiple sclerosis model	Mouse	50–200 mg/kg	Modulated immune cells; neuroprotective.	[76]
	In vivo	DMH-induced preneoplastic colorectal lesions	Rat	Isoliquiritigenin-rich extract	Reduced preneoplastic lesions by 41.6%.	[63]
	In vitro	Pancreatic cancer cells	Human	12.5–25 µM	Induced apoptosis through autophagy blockade mediated by p38 MAPK; synergistic with chemotherapies.	[77]
Liquiritigenin	In vivo	Collagen-induced arthritis	Mouse	100–300 mg/kg	Reduced TNF-α, IL-6 via NF-κB inhibition.	[78]
	In vitro	IL-1β-stimulated chondrocytes	Human	20–40 µM	Reduced MMPs; protected cartilage.	[79]
	In vivo	Rheumatoid arthritis with cardiac involvement	Mouse	20 mg/kg	Improved joint and cardiac function; downregulated pro-inflammatory cytokines, MMP-3/MMP-13, and TGF-β1/Smad2/3 signaling.	[80]
	In vitro	Dendritic and T cells	Human	10–50 µM	Increased cAMP via adenylyl cyclase modulation; suppressed pro-inflammatory cytokines and shifted T-cell polarization.	[81]
	In vivo	Alzheimer’s disease model	Mouse	30 mg/kg	Shifted microglia to M2 phenotype; reduced NLRP3 and cleaved caspase-1; improved cognition.	[82]
	In vivo	Arsenic trioxide-induced liver injury	Mouse	20–40 mg/kg	Reduced oxidative stress and inflammation via mTOR-mediated autophagy activation.	[83]
Guttiferone E	In vivo	Colon carcinogenesis (DMH)	Rat	12–48 mg/kg p.o.	Reduced COX-2, PCNA expression.	[88]
	In vivo	NSCLC xenograft	Mouse	10 mg/kg i.p.	Reduced mTOR, SIRT1, COX-2; tumor and inflammation inhibition.	[89]
Oblongifolin B	-	-	-	-	No specific anti-inflammatory study detailed.	-
β-amyrin	In vivo	CCl_4_-induced hepatic fibrosis	Mouse	25, 50 mg/kg oral, 3×/week for 6 weeks	Reduced TNF-α, IL-1β, IL-6; attenuated collagen deposition and fibrotic markers; modulated apoptosis-related proteins.	[91]
	In vitro	LPS/IFN-γ-stimulated microglial cells	Murine	12, 25, 50 µM	Suppressed pro-inflammatory cytokines and enzymes; promoted M2 polarization; no cytotoxicity.	[92]
	In vivo	Nickel chloride-induced colon cancer	Rat	2.5 mg/kg oral	Reduced inflammatory and oxidative markers; improved antioxidant enzymes; synergistic with β-sitosterol and epiafzelechin.	[93]
	In vivo	Bleomycin-induced pulmonary fibrosis	Mouse	20, 40, 80 mg/kg oral	Reduced pulmonary inflammation, oxidative stress, collagen deposition; improved lung function.	[94]

## Data Availability

The data supporting the findings of this study are derived from previously published articles, which are cited throughout the manuscript. No new datasets were generated during the current study.

## Abbreviations

The following abbreviations are used in this manuscript:

Aβ	Amyloid Beta
ADME	Absorption, distribution, metabolism, and excretion
AKI	Acute kidney injury
Arg-1	Arginase-1
ARE	Antioxidant response element
BAX	Bcl-2-associated X protein
BBB	Blood–brain barrier
Bcl-2	B-cell lymphoma 2
BRP	Brazilian red propolis
BV2	Microglial BV2 cell line
CAT	Catalase
CD	Cluster of differentiation
COX-2	Cyclooxygenase-2
CXCL1	C-X-C motif chemokine ligand 1
CXCR3	C-X-C chemokine receptor type 3
cAMP	Cyclic adenosine monophosphate
CYP51	Sterol 14-alpha-demethylase
DMH	1,2-dimethylhydrazine
DOX	Doxorubicin
EGF	Epidermal growth gactor
EMT	Epithelial–mesenchymal transition
ER	Estrogen receptor
ERK	Extracellular signal-regulated kinase
FOXO3a	Forkhead box O3a
FOXP3	Forkhead box P3
G6PD	Glucose-6-phosphate dehydrogenase
GPX4	Glutathione peroxidase 4
GR	Glutathione reductase
GSH	Glutathione
HO-1	Heme oxygenase 1
HSFs	Human skin fibroblasts
IC50	Half maximal inhibitory concentration
ICAM-1	Intercellular adhesion molecule-1
IFN	Interferon
IFN-α	Interferon-alpha
IFN-β	Interferon-beta
IFN-γ	Interferon-gamma
IKK	IκB kinase
IL-1β	Interleukin 1 beta
IL-6	Interleukin 6
IRF1	Interferon regulatory factor 1
JAK2	Janus kinase 2
JNK	c-Jun N-terminal kinase
Keap-1	Kelch-like ECH-associated protein 1
LC3II/I	Microtubule-associated proteins 1A/1B light chain 3B (form II/I)
LPS	Lipopolysaccharide
M1/M2	Macrophage phenotypes: M1 (pro-inflammatory), M2 (anti-inflammatory)
MCAO	Middle cerebral artery occlusion
MCP-1	Monocyte chemoattractant protein-1
MAPK	Mitogen-activated protein kinase
MDA	Malondialdehyde
MDCK	Madin–Darby canine kidney
MMP-2	Matrix metallopeptidase 2
MRSA	Methicillin-resistant *Staphylococcus Aureus*
mTOR	Mammalian target of rapamycin
NLRP3	NLR family pyrin domain containing 3
NF-κB	Nuclear factor κB (nuclear factor kappa-light-chain-enhancer of activated B cells)
NO	Nitric oxide
NQO1	NAD(P)H quinone dehydrogenase 1
Nrf2	Nuclear factor erythroid 2–related factor 2
NSCLC	Non-small-cell lung cancer
OGD	Oxygen-glucose deprivation
P62	Sequestosome 1
PASI	Psoriasis area and severity index
PCNA	Proliferating cell nuclear antigen
PD-L1	Programmed death-ligand 1
PGE2	Prostaglandin E2
PGES	Prostaglandin E synthase
PI3K	Phosphoinositide 3-kinase
PTGS2	Prostaglandin-endoperoxide synthase 2
P-gp	P-glycoprotein
ROS	Reactive oxygen species
RORγt	RAR-related orphan receptor gamma
SIRT3	Sirtuin 3
Smad2/3	SMAD family member 2 and 3
SOD	Superoxide dismutase
STAT	Signal transducer and activator of transcription
TAC	Transverse aortic constriction
TGF-β1	Transforming growth factor beta 1
TLR4	Toll-like receptor 4
TNF-α	Tumor necrosis factor alpha
TR	Trypanothione reductase
TSLP	Thymic stromal lymphopoietin
VEGF	Vascular endothelial growth factor
ZO-1	Zonula occludens-1

## References

[1] Freires I.A., De Alencar S.M., Rosalen P.L. (2016). A Pharmacological Perspective on the Use of Brazilian Red Propolis and Its Isolated Compounds against Human Diseases. Eur. J. Med. Chem..

[2] Rufatto L.C., Luchtenberg P., Garcia C., Thomassigny C., Bouttier S., Henriques J.A.P., Roesch-Ely M., Dumas F., Moura S. (2018). Brazilian Red Propolis: Chemical Composition and Antibacterial Activity Determined Using Bioguided Fractionation. Microbiol. Res..

[3] Silva B.B., Rosalen P.L., Cury J.A., Ikegaki M., Souza V.C., Esteves A., Alencar S.M. (2008). Chemical Composition and Botanical Origin of Red Propolis, a New Type of Brazilian Propolis. Evid.-Based Complement. Altern. Med..

[4] Bueno-Silva B., Marsola A., Ikegaki M., Alencar S.M., Rosalen P.L. (2017). The Effect of Seasons on Brazilian Red Propolis and Its Botanical Source: Chemical Composition and Antibacterial Activity. Nat. Prod. Res..

[5] Ccana-Ccapatinta G.V., Mejía J.A.A., Tanimoto M.H., Groppo M., de Carvalho J.C.A.S., Bastos J.K. (2020). *Dalbergia ecastaphyllum* (L.) Taub. and *Symphonia globulifera* L.f.: The Botanical Sources of Isoflavonoids and Benzophenones in Brazilian Red Propolis. Molecules.

[6] Pemmari A., Moilanen E. (2024). Macrophage and Chondrocyte Phenotypes in Inflammation. Basic. Clin. Pharmacol. Toxicol..

[7] Pan X., Jiang S., Zhang X., Wang Z., Wang X., Cao L., Xiao W. (2024). Recent Strategies in Target Identification of Natural Products: Exploring Applications in Chronic Inflammation and Beyond. Br. J. Pharmacol..

[8] Aldana-Mejía J.A., Ccana-Ccapatinta G.V., Ribeiro V.P., Arruda C., Veneziani R.C.S., Ambrósio S.R., Bastos J.K. (2021). A Validated HPLC-UV Method for the Analysis of Phenolic Compounds in Brazilian Red Propolis and *Dalbergia ecastaphyllum*. J. Pharm. Biomed. Anal..

[9] Bueno-Silva B., Alencar S.M., Koo H., Ikegaki M., Silva G.V.J., Napimoga M.H., Rosalen P.L. (2013). Anti-Inflammatory and Antimicrobial Evaluation of Neovestitol and Vestitol Isolated from Brazilian Red Propolis. J. Agric. Food Chem..

[10] de Moraes Porto I.C.C., de Barros Rocha A.B., Ferreira I.I.S., de Barros B.M., Ávila E.C., da Silva M.C., de Oliveira M.P.S., Lôbo T.d.L.G.F., Oliveira J.M.d.S., do Nascimento T.G. (2021). Polyphenols and Brazilian Red Propolis Incorporated into a Total-Etching Adhesive System Help in Maintaining Bonding Durability. Heliyon.

[11] de Freitas M.C.D., de Miranda M.B., de Oliveira D.T., Vieira-Filho S.A., Caligiorne R.B., de Figueiredo S.M. (2018). Biological Activities of Red Propolis: A Review. Recent. Pat. Endocr. Metab. Immune Drug Discov..

[12] Daugsch A., Moraes C.S., Fort P., Park Y.K. (2007). Brazilian Red Propolis—Chemical Composition and Botanical Origin. eCAM.

[13] Lima Cavendish R., de Souza Santos J., Belo Neto R., Oliveira Paixão A., Valéria Oliveira J., Divino de Araujo E., Berretta e Silva A.A., Maria Thomazzi S., Cordeiro Cardoso J., Zanardo Gomes M. (2015). Antinociceptive and Anti-Inflammatory Effects of Brazilian Red Propolis Extract and Formononetin in Rodents. J. Ethnopharmacol..

[14] Barbosa R.A., Nunes T.L.G.M., Nunes T.L.G.M., Paixão A.O.D., Neto R.B., Moura S., Albuquerque Junior R.L.C., Cândido E.A.F., Padilha F.F., Quintans-Júnior L.J. (2016). Hydroalcoholic Extract of Red Propolis Promotes Functional Recovery and Axon Repair after Sciatic Nerve Injury in Rats. Pharm. Biol..

[15] Xu H.T., Zheng Q., Tai Z.G., Jiang W.C., Xie S.Q., Luo Y., Fei X.Y., Luo Y., Ma X., Kuai L. (2024). Formononetin Attenuates Psoriasiform Inflammation by Regulating Interferon Signaling Pathway. Phytomedicine.

[16] Xu L., Zhou S., Li J., Yu W., Gao W., Luo H., Fang X. (2025). The Anti-Inflammatory Effects of Formononetin, an Active Constituent of Pueraria Montana Var. Lobata, via Modulation of Macrophage Autophagy and Polarization. Molecules.

[17] Liu Z., Wang R.H., Wang K.H. (2025). Formononetin Ameliorates Polycystic Ovary Syndrome through Suppressing NLRP3 Inflammasome. Mol. Med..

[18] Zhang Y., Deng J., Chen T., Liu S., Tang Y., Zhao J.R., Guo Z., Zhang W. (2024). Formononetin Alleviates No Reflow after Myocardial Ischemia-Reperfusion via Modulation of Gut Microbiota to Inhibit Inflammation. Life Sci..

[19] Yang M., Yang Z., Huang X., Li X., Chou F., Zeng S. (2024). Formononetin Alleviates Thermal Injury-Induced Skin Fibroblast Apoptosis and Promotes Cell Proliferation and Migration. Burns.

[20] Chen J., Cai Y., Wei D., Cao L., He Q., Zhang Y. (2024). Formononetin Inhibits Neuroinflammation in BV2 Microglia Induced by Glucose and Oxygen Deprivation Reperfusion through TLR4/NF-ΚB Signaling Pathway. Brain Res..

[21] de Mendonça M.A.A., Ribeiro A.R.S., de Lima A.K., Bezerra G.B., Pinheiro M.S., de Albuquerque-Júnior R.L.C., Gomes M.Z., Padilha F.F., Thomazzi S.M., Novellino E. (2020). Red Propolis and Its Dyslipidemic Regulator Formononetin: Evaluation of Antioxidant Activity and Gastroprotective Effects in Rat Model of Gastric Ulcer. Nutrients.

[22] Jeong E.J., Jia X., Hu M. (2005). Disposition of Formononetin via Enteric Recycling: Metabolism and Excretion in Mouse Intestinal Perfusion and Caco-2 Cell Models. Mol. Pharm..

[23] Luo L.-Y., Fan M.-X., Zhao H.-Y., Li M.-X., Wu X., Gao W.-Y. (2018). Pharmacokinetics and Bioavailability of the Isoflavones Formononetin and Ononin and Their in Vitro Absorption in Ussing Chamber and Caco-2 Cell Models. J. Agric. Food Chem..

[24] Li C., Li G., Gao Y., Sun C., Wang X. (2016). A 90-Day Subchronic Toxicity Study with Sodium Formononetin-3’-Sulphonate (Sul-F) Delivered to Dogs via Intravenous Administration. Regul. Toxicol. Pharmacol..

[25] Wang D., Zheng C., Chen B., Ma S. (2024). Biochanin A Induces Apoptosis in MCF-7 Breast Cancer Cells through Mitochondrial Pathway and Pi3K/AKT Inhibition. Cell Biochem. Funct..

[26] Wu P.S., Yen J.H., Chen P.Y., Wu M.J. (2025). Molecular Mechanisms of Biochanin A in AML Cells: Apoptosis Induction and Pathway-Specific Regulation in U937 and THP-1. Int. J. Mol. Sci..

[27] Andugulapati S.B., Gourishetti K., Tirunavalli S.K., Shaikh T.B., Sistla R. (2020). Biochanin-A Ameliorates Pulmonary Fibrosis by Suppressing the TGF-β Mediated EMT, Myofibroblasts Differentiation and Collagen Deposition in in Vitro and in Vivo Systems. Phytomedicine.

[28] Zhang S., Niu Y., Yang Z., Zhang Y., Guo Q., Yang Y., Zhou X., Ding Y., Liu C. (2020). Biochanin A Alleviates Gingival Inflammation and Alveolar Bone Loss in Rats with Experimental Periodontitis. Exp. Ther. Med..

[29] Walvekar K.P., Tirunavalli S.K., Eedara A.C., Chandra Y., Kuncha M., B.R.Kumar A., Sistla R., Andugulapati S.B., Chilaka S. (2024). Biochanin A Ameliorates Imiquimod-Induced Psoriasis-Like Skin Inflammation in Mice by Modulating the NF-ΚB and MAPK Signaling Pathways. Inflammation.

[30] Lv Y., Xu Y., Liu S., Zeng X., Yang B. (2024). Biochanin A Attenuates Psoriasiform Inflammation by Regulating Nrf2/HO-1 Pathway Activation and Attenuating Inflammatory Signalling. Cell Biochem. Biophys..

[31] Feng Z., Zhang N., Bai J., Lin Q.Y., Xie Y., Xia Y.-L. (2024). Biochanin A Inhibits Cardiac Hypertrophy and Fibrosis in Vivo and in Vitro. Biomed. Pharmacother..

[32] Mahajan U.B., Goyal S. (2025). Biochanin A Mitigates Oxidative Stress and Inflammation in Diabetic Myocardial Infarction: Insights From a Streptozotocin and Isoproterenol Rat Model. Cureus.

[33] Deng B., Wang K., He H., Xu M., Li J., He P., Liu Y., Ma J., Zhang J., Dong W. (2025). Biochanin A Mitigates Colitis by Inhibiting Ferroptosis-Mediated Intestinal Barrier Dysfunction, Oxidative Stress, and Inflammation via the JAK2/STAT3 Signaling Pathway. Phytomedicine.

[34] Ram C., Gairola S., Syed A.M., Kulhari U., Kundu S., Mugale M.N., Murty U.S., Sahu B.D. (2022). Biochanin A Alleviates Unilateral Ureteral Obstruction-Induced Renal Interstitial Fibrosis and Inflammation by Inhibiting the TGF-Β1/Smad2/3 and NF-KB/NLRP3 Signaling Axis in Mice. Life Sci..

[35] Li X., Fu J., Guan M., Shi H., Pan W., Lou X. (2024). Biochanin A Attenuates Spinal Cord Injury in Rats during Early Stages by Inhibiting Oxidative Stress and Inflammasome Activation. Neural Regen. Res..

[36] Thakkar A.B., Subramanian R.B., Thakkar S.S., Thakkar V.R., Thakor P. (2024). Biochanin A—A G6PD Inhibitor: In Silico and in Vitro Studies in Non-Small Cell Lung Cancer Cells (A549). Toxicol. Vitr..

[37] Batista C.M., Alves A.V.F., Queiroz L.A., Lima B.S., Filho R.N.P., Araújo A.A.S., de Albuquerque Júnior R.L.C., Cardoso J.C. (2018). The Photoprotective and Anti-Inflammatory Activity of Red Propolis Extract in Rats. J. Photochem. Photobiol. B.

[38] Barbosa Bezerra G., de Menezes de Souza L., dos Santos A.S., de Almeida G.K.M., Souza M.T.S., Santos S.L., Aparecido Camargo E., dos Santos Lima B., de Souza Araújo A.A., Cardoso J.C. (2017). Hydroalcoholic Extract of Brazilian Red Propolis Exerts Protective Effects on Acetic Acid-Induced Ulcerative Colitis in a Rodent Model. Biomed. Pharmacother..

[39] Tan Y., Zhang X., Cheang W.S. (2022). Isoflavones Daidzin and Daidzein Inhibit Lipopolysaccharide-Induced Inflammation in RAW264.7 Macrophages. Chin. Med..

[40] Yu Z., Yang L., Deng S., Liang M. (2020). Daidzein Ameliorates LPS-Induced Hepatocyte Injury by Inhibiting Inflammation and Oxidative Stress. Eur. J. Pharmacol..

[41] Li H., Zhang M., Wang Y., Gong K., Yan T., Wang D., Meng X., Yang X., Chen Y., Han J. (2022). Daidzein Alleviates Doxorubicin-Induced Heart Failure via the SIRT3/FOXO3a Signaling Pathway. Food Funct..

[42] Xie K., Li Y., He G., Zhao X., Chen D., Yu B., Luo Y., Mao X., Huang Z., Yu J. (2022). Daidzein Supplementation Improved Fecundity in Sows via Modulation of Ovarian Oxidative Stress and Inflammation. J. Nutr. Biochem..

[43] Márquez-Flores Y.K., Martínez-Galero E., Correa-Basurto J., Sixto-López Y., Villegas I., Rosillo M., Cárdeno A., Alarcón-de-la-Lastra C. (2024). Daidzein and Equol: Ex Vivo and In Silico Approaches Targeting COX-2, INOS, and the Canonical Inflammasome Signaling Pathway. Pharmaceuticals.

[44] Liu Z.M., Chen B., Li S., Li G., Zhang D., Ho S.C., Chen Y.M., Ma J., Qi H., Ling W.H. (2020). Effect of Whole Soy and Isoflavones Daidzein on Bone Turnover and Inflammatory Markers: A 6-Month Double-Blind, Randomized Controlled Trial in Chinese Postmenopausal Women Who Are Equol Producers. Ther. Adv. Endocrinol. Metab..

[45] Özdemir A.Y., Çetin E.A., Novotný J., Rudajev V. (2025). Daidzein Effectively Mitigates Amyloid-β-Induced Damage in SH-SY5Y Neuroblastoma Cells and C6 Glioma Cells. Biomed. Pharmacother..

[46] Guru A., Sudhakaran G., Velayutham M., Murugan R., Pachaiappan R., Mothana R.A., Noman O.M., Juliet A., Arockiaraj J. (2022). Daidzein Normalized Gentamicin-Induced Nephrotoxicity and Associated pro-Inflammatory Cytokines in MDCK and Zebrafish: Possible Mechanism of Nephroprotection. Comp. Biochem. Physiol. Part C Toxicol. Pharmacol..

[47] Kassab R.B., Elhenawy A.A., AbdulrahmanTheyab, Hawsawi Y.M., Al-Amer O.M., Oyouni A.A.A., Habotta O.A., Althagafi H.A., Alharthi F., Lokman M.S. (2023). Modulation of Inflammatory, Oxidative, and Apoptotic Stresses Mediates the Renoprotective Effect of Daidzein against Glycerol-Induced Acute Kidney Injury in Rats. Environ. Sci. Pollut. Res. Int..

[48] Karale S., Kamath J.V. (2017). Effect of Daidzein on Cisplatin-Induced Hematotoxicity and Hepatotoxicity in Experimental Rats. Indian. J. Pharmacol..

[49] Laddha A.P., Murugesan S., Kulkarni Y.A. (2022). In-Vivo and in-Silico Toxicity Studies of Daidzein: An Isoflavone from Soy. Drug Chem. Toxicol..

[50] Faria W.C.S., Martinelli R.C., Arcas A.S., Da Silva Junior I.F., Colodel E.M., Cavenaghi D., Oliveira A.P., Barros W.M. (2018). Acute and Subacute Toxicity Study on Dietary Supplementation with Soy Isoflavones in Wistar Rats. Curr. Nutr. Food Sci..

[51] Yang X., Pan Y., Cai L., Wang W., Zhai X., Zhang Y., Wu Q., Chen J., Zhang C., Wang Y. (2024). Calycosin Ameliorates Neuroinflammation via TLR4-Mediated Signal Following Cerebral Ischemia/Reperfusion Injury in Vivo and in Vitro. J. Inflamm. Res..

[52] Liu X., Shao Y., Zhang X., Ji X., Xie M., Liu H. (2021). Calycosin Attenuates Pulmonary Fibrosis by the Epithelial-Mesenchymal Transition Repression upon Inhibiting the AKT/GSK3β/β-Catenin Signaling Pathway. Acta Histochem..

[53] Liu H., Bai X., Wei W., Li Z., Zhang Z., Tan W., Wei B., Zhao H., Jiao Y. (2022). Calycosin Ameliorates Bleomycin-Induced Pulmonary Fibrosis via Suppressing Oxidative Stress, Apoptosis, and Enhancing Autophagy. Evid.-Based Complement. Altern. Med..

[54] Ma X., Deng G., Tian N., Wang H., Zhao H., Kuai L., Luo Y., Gao C., Ding X., Li B. (2024). Calycosin Enhances Treg Differentiation for Alleviating Skin Inflammation in Atopic Dermatitis. J. Ethnopharmacol..

[55] Peng H., Jin L., Zhang Q., Shen Y., Wang Z., Zhou F., Yu Q. (2022). Calycosin Improves Intestinal Mucosal Barrier Function after Gastrectomy in Rats through Alleviating Bacterial Translocation, Inflammation, and Oxidative Stress. Evid.-Based Complement. Altern. Med..

[56] Wang X., Li W., Zhang Y., Sun Q., Cao J., Tan N.N., Yang S., Lu L., Zhang Q., Wei P. (2022). Calycosin as a Novel PI3K Activator Reduces Inflammation and Fibrosis in Heart Failure Through AKT–IKK/STAT3 Axis. Front. Pharmacol..

[57] Xu S., Huang P., Yang J., Du H., Wan H., He Y. (2023). Calycosin Alleviates Cerebral Ischemia/Reperfusion Injury by Repressing Autophagy via STAT3/FOXO3a Signaling Pathway. Phytomedicine.

[58] Shi X., Jie L., Wu P., Zhang N., Mao J., Wang P., Yin S. (2022). Calycosin Mitigates Chondrocyte Inflammation and Apoptosis by Inhibiting the PI3K/AKT and NF-ΚB Pathways. J. Ethnopharmacol..

[59] Huang C., Xue L.F., Hu B., Liu H.H., Huang S.B., Khan S., Meng Y. (2021). Calycosin-Loaded Nanoliposomes as Potential Nanoplatforms for Treatment of Diabetic Nephropathy through Regulation of Mitochondrial Respiratory Function. J. Nanobiotechnol..

[60] Kim J.H., Kang D.M., Cho Y.J., Hyun J.W., Ahn M.J. (2022). Medicarpin Increases Antioxidant Genes by Inducing NRF2 Transcriptional Level in HeLa Cells. Antioxidants.

[61] Boeing T., Mejía J.A.A., Ccana-Ccapatinta G.V., Mariott M., de Cássia Melo Vilhena de Andrade Fonseca Da Silva R., de Souza P., Mariano L.N.B., Oliveira G.R., da Rocha I.M., da Costa G.A. (2021). The Gastroprotective Effect of Red Propolis Extract from Northeastern Brazil and the Role of Its Isolated Compounds. J. Ethnopharmacol..

[62] Chen Y., Yin L., Hao M., Xu W., Gao J., Sun Y., Wang Q., Chen S., Liang Y., Guo R. (2023). Medicarpin Induces G1 Arrest and Mitochondria-Mediated Intrinsic Apoptotic Pathway in Bladder Cancer Cells. Acta Pharm..

[63] Squarisi I.S., Ribeiro V.P., Ribeiro A.B., de Souza L.T.M., de Melo Junqueira M., de Oliveira K.M., Hayot G., Dickmeis T., Bastos J.K., Veneziani R.C.S. (2024). Development of a Benzophenone-Free Red Propolis Extract and Evaluation of Its Efficacy against Colon Carcinogenesis. Pharmaceuticals.

[64] Williams D., Perry D., Carraway J., Simpson S., Uwamariya P., Christian O.E. (2021). Antigonococcal Activity of (+)-Medicarpin. ACS Omega.

[65] Aldana-Mejía J.A., Ccana-Ccapatinta G.V., Squarisi I.S., Nascimento S., Tanimoto M.H., Ribeiro V.P., Arruda C., Nicolella H., Esperandim T., Ribeiro A.B. (2021). Nonclinical Toxicological Studies of Brazilian Red Propolis and Its Primary Botanical Source *Dalbergia ecastaphyllum*. Chem. Res. Toxicol..

[66] Franchin M., Colón D.F., Da Cunha M.G., Castanheira F.V.S., Saraiva A.L.L., Bueno-Silva B., Alencar S.M., Cunha T.M., Rosalen P.L. (2016). Neovestitol, an Isoflavonoid Isolated from Brazilian Red Propolis, Reduces Acute and Chronic Inflammation: Involvement of Nitric Oxide and IL-6. Sci. Rep..

[67] Bueno-Silva B., Rosalen P.L., Alencar S.M., Mayer M.P.A. (2020). Vestitol Drives LPS-Activated Macrophages into M2 Phenotype through Modulation of NF-ΚB Pathway. Int. Immunopharmacol..

[68] Bueno-silva B., Bueno M.R., Kawamoto D., Casarin R.C., Pingueiro J.M.S., Alencar S.M., Rosalen P.L., Mayer M.P.A. (2022). Anti-Inflammatory Effects of (3S)-Vestitol on Peritoneal Macrophages. Pharmaceuticals.

[69] Nani B.D., Franchin M., Lazarini J.G., Freires I.A., da Cunha M.G., Bueno-Silva B., de Alencar S.M., Murata R.M., Rosalen P.L. (2018). Isoflavonoids from Brazilian Red Propolis Down-Regulate the Expression of Cancer-Related Target Proteins: A Pharmacogenomic Analysis. Phytother. Res..

[70] Aldana-Mejía J.A., Ribeiro V.P., Meepagala K.M., Bastos J.K., Ross S.A. (2025). Bioactive Metabolites of Brazilian Red Propolis: Cytotoxic, Antimalarial, and Antimicrobial Properties. Fitoterapia.

[71] Da Silva R.O., Andrade V.M., Bullé Rêgo E.S., Azevedo Dória G.A., dos Santos Lima B., Da Silva F.A., De Souza Araújo A.A., De Albuquerque Júnior R.L.C., Cordeiro Cardoso J., Zanardo Gomes M. (2015). Acute and Sub-Acute Oral Toxicity of Brazilian Red Propolis in Rats. J. Ethnopharmacol..

[72] Dutra R.P., de Sousa M.M., Mignoni M.S.P.M., de Oliveira K.G.M., Pereira E.B., Figueredo A.S., da Costa A.A.C., Dias T.G., Vasconcelos C.C., Silva L.A. (2023). Brazilian Amazon Red Propolis: Leishmanicidal Activity and Chemical Composition of a New Variety of Red Propolis. Metabolites.

[73] Oldoni T.L.C., Cabral I.S.R., D’Arce M.A.B.R., Rosalen P.L., Ikegaki M., Nascimento A.M., Alencar S.M. (2011). Isolation and Analysis of Bioactive Isoflavonoids and Chalcone from a New Type of Brazilian Propolis. Sep. Purif. Technol..

[74] Chen Z., Ding W., Yang X., Lu T., Liu Y. (2024). Isoliquiritigenin, a Potential Therapeutic Agent for Treatment of Inflammation-Associated Diseases. J. Ethnopharmacol..

[75] Huang X., Shi Y., Chen H., Le R., Gong X., Xu K., Zhu Q., Shen F., Chen Z., Gu X. (2020). Isoliquiritigenin Prevents Hyperglycemia-Induced Renal Injuries by Inhibiting Inflammation and Oxidative Stress via SIRT1-Dependent Mechanism. Cell Death Dis..

[76] Feng R., Meng T., Zhao X., Yu W., Li H., Wang Z., Chen J., Yang C. (2024). Isoliquiritigenin Reduces Experimental Autoimmune Prostatitis by Facilitating Nrf2 Activation and Suppressing the NLRP3 Inflammasome Pathway. Mol. Immunol..

[77] Zhang Z., Chen W.-q., Zhang S.-q., Bai J.-x., Liu B., Yung K.K.-L., Ko J.K.-S. (2022). Isoliquiritigenin Inhibits Pancreatic Cancer Progression through Blockade of P38 MAPK-Regulated Autophagy. Phytomedicine.

[78] Babu V., Kapkoti D.S., Binwal M., Bhakuni R.S., Shanker K., Singh M., Tandon S., Mugale M.N., Kumar N., Bawankule D.U. (2023). Liquiritigenin, Isoliquiritigenin Rich Extract of Glycyrrhiza Glabra Roots Attenuates Inflammation in Macrophages and Collagen-Induced Arthritis in Rats. Inflammopharmacology.

[79] Tu C., Ma Y., Song M., Yan J., Xiao Y., Wu H. (2019). Liquiritigenin Inhibits IL-1β-Induced Inflammation and Cartilage Matrix Degradation in Rat Chondrocytes. Eur. J. Pharmacol..

[80] Ning X., Ni Y., Cao J., Zhang H. (2023). Liquiritigenin Attenuated Collagen-Induced Arthritis and Cardiac Complication via Inflammation and Fibrosis Inhibition in Mice. Chem Pharm Bull.

[81] Qin M., Guo A., Li F., Zhang F., Bi M., Zhang Y., Zhu W. (2021). Liquiritigenin Enhances Cyclic Adenosine Monophosphate Production to Mitigate Inflammation in Dendritic Cells. Int. J. Immunopathol. Pharmacol..

[82] Du Y., Luo M., Du Y., Xu M., Yao Q., Wang K., He G. (2021). Liquiritigenin Decreases Aβ Levels and Ameliorates Cognitive Decline by Regulating Microglia M1/M2 Transformation in AD Mice. Neurotox. Res..

[83] Zhang M., Xue Y., Zheng B., Li L., Chu X., Zhao Y., Wu Y., Zhang J., Han X., Wu Z. (2021). Liquiritigenin Protects against Arsenic Trioxide-Induced Liver Injury by Inhibiting Oxidative Stress and Enhancing MTOR-Mediated Autophagy. Biomed. Pharmacother..

[84] Song Z., Zhang Y., Zhang H., Rajendran R.S., Wang R., Hsiao C.D., Li J., Xia Q., Liu K. (2020). Isoliquiritigenin Triggers Developmental Toxicity and Oxidative Stress–Mediated Apoptosis in Zebrafish Embryos/Larvae via Nrf2-HO1/JNK-ERK/Mitochondrion Pathway. Chemosphere.

[85] Yang K., Ganesan K., Gao F., Xie C., Chen J. (2024). Subacute Toxicity of Isoliquiritigenin-Zein Phosphatidylcholine Nanoparticles on Biochemical, Hematological, and Histopathological Parameters in Sprague-Dawley Rats. Explor. Drug Sci..

[86] Fasolo D., Bergold A.M., von Poser G., Teixeira H.F. (2016). Determination of Benzophenones in Lipophilic Extract of Brazilian Red Propolis, Nanotechnology-Based Product and Porcine Skin and Mucosa: Analytical and Bioanalytical Assays. J. Pharm. Biomed. Anal..

[87] Pires L.M., Santos M.F.C., Figueiredo L.R., Faleiros R., Badoco F.R., Silva K.B., Ambrósio S.R., Bastos J.K., SANTOS R.A.A., Veneziani R.C.S. (2024). Polyprenylated Benzophenones from Brazilian Red Propolis: Analytical Characterization and Anticancer Activity. Chem. Biodivers..

[88] De Freitas K.S., Da Silva L.H.D., Squarisi I.S., De Souza Oliveira L.T., Ribeiro A.B., Alves B.S., Esperandim T.R., De Melo M.R.S., Ozelin S.D., Lemes D.C. (2022). Red Propolis Exhibits Chemopreventive Effect Associated with Antiproliferative and Anti-Inflammatory Activities. Toxicol. Res..

[89] Nathani A., Khan I., Tanimoto M.H., Mejía J.A.A., DE Miranda A.M., Rishi A., Dev S., Bastos J.K., Singh M. (2024). Antitumor Potential of Guttiferone E Combined With Carboplatin Against Osimertinib-Resistant H1975 Lung Cancer Through Apoptosis. Anticancer. Res..

[90] Ripari N., Lopes E.C., Francisco Á.F., de Almeida J.F.S., da Silva Honorio M., Júnior A.F., de Mattos Fontes R., Tanimoto M.H., de Azevedo Calderon L., Bastos J.K. (2025). Guttiferone E from Brazilian Red Propolis Inhibited Wound-Isolated Methicillin-Resistant Staphylococcus Aureus and Enhanced the Bactericidal Action of Suppressed Macrophages. Phytomedicine.

[91] Thirupathi A., Silveira P.C., Nesi R.T., Pinho R.A. (2017). β-Amyrin, a Pentacyclic Triterpene, Exhibits Anti-Fibrotic, Anti-Inflammatory, and Anti-Apoptotic Effects on Dimethyl Nitrosamine-Induced Hepatic Fibrosis in Male Rats. Hum. Exp. Toxicol..

[92] Askari V.R., Fereydouni N., Baradaran Rahimi V., Askari N., Sahebkar A.H., Rahmanian-Devin P., Samzadeh-Kermani A. (2018). β-Amyrin, the Cannabinoid Receptors Agonist, Abrogates Mice Brain Microglial Cells Inflammation Induced by Lipopolysaccharide/Interferon-γ and Regulates Mφ1/Mφ2 Balances. Biomed. Pharmacother..

[93] Zahid S., Malik A., Waqar S., Zahid F., Tariq N., Khawaja A.I., Safir W., Gulzar F., Iqbal J., Ali Q. (2023). Countenance and Implication of Β-Sitosterol, Β-Amyrin and Epiafzelechin in Nickel Exposed Rat: In-Silico and in-Vivo Approach. Sci. Rep..

[94] Cai Z., Liu J., Bian H., Cai J. (2023). β-Amyrin Ameliorates Pulmonary Fibrosis by Inhibiting Inflammatory Response and Oxidative Stress in Mice. Pak. J. Pharm. Sci..

[95] Lissin L.W., Oka R., Lakshmi S., Cooke J.P. (2004). Isoflavones Improve Vascular Reactivity in Post-Menopausal Women with Hypercholesterolemia. Vasc. Med..

[96] Abshirini M., Omidian M., Kord-Varkaneh H. (2020). Effect of Soy Protein Containing Isoflavones on Endothelial and Vascular Function in Postmenopausal Women: A Systematic Review and Meta-Analysis of Randomized Controlled Trials. Menopause.

